# “I must be in control!”: the role of irrational beliefs, challenge and threat, and self-control, in the mental health of employees

**DOI:** 10.3389/fpsyg.2026.1633485

**Published:** 2026-07-31

**Authors:** Paul C. Mansell, Andrew G. Wood, Martin J. Turner

**Affiliations:** 1 School of Health, Education, Policing and Sciences, University of Staffordshire, Stoke-on-Trent, United Kingdom; 2 Faculty of Health and Education, Manchester Metropolitan University, Manchester, United Kingdom

**Keywords:** appraisal, core beliefs, distress, REBT, SEM

## Abstract

**Introduction:**

In the working population, the prevalence of poor mental health continues to increase. Employees are often confronted by workplace stressors, but it is not the experience of stressors alone which determines poor mental health. There are various psychological factors which may increase risks to mental health. The present study investigates how specific psychological factors, namely irrational beliefs, challenge and threat evaluations and self-control, may interact to influence employee mental health indicated by symptoms of anxiety, depression, and anger.

**Methods:**

Three hundred and fifty-one adults (*M*age = 39.25 years, *SD* = 11.85) from a wide range of occupations completed a questionnaire pack.

**Results:**

Data were modelled using path analysis, indicating that irrational beliefs may undermine employee mental health through threat evaluations and self-control, such that stronger irrational beliefs, higher threat, and poorer self-control were associated with poorer mental health markers. In addition, irrational beliefs were not directly related to self-control, only indirectly through challenge and threat.

**Discussion:**

It would appear that, in support of previous research, challenge and threat evaluations are a risk factor for poor mental health. Accordingly, reducing threat evaluations and developing challenge evaluations may be important in enhancing employee mental health.

## Introduction

One in six adults in the UK are said to have experienced a common mental disorder such as anxiety or depression in the past week (Baker, 2020). The prevalence of anxiety and depression among adults continues to grow, with depression identified as potentially the leading cause of illness worldwide by 2030 (Hoying et al., 2020). In the workplace, two-thirds of employees have reported feeling stressed or anxious about their job over the course of a 12-month period, with this number increasing to 75% for those under 35 (ACAS, 2019). Whether psychological distress is experienced at a subclinical or clinical level, both are common and can impair performance at work (LaMontagne et al., 2014). Indeed, due to the stigma of disclosing poor mental health in the workplace (Zamir et al., 2022), many more employees may be experiencing psychological distress at a subclinical level. Consequently, the experience of high stress will likely result in reduced productivity (Halkos and Bousinakis, 2010) and increased absence from work (Sanchez-Gomez et al., 2021). However, not all individuals who experience high stress will experience poor mental health, and it is important to understand the factors that can influence an individual’s mental health when experiencing stress in the workplace.

Employees face a multitude of ongoing workplace demands which may be exacerbated by increasingly complex work processes. These demands can be categorized into four sub-groups including task demands (e.g., job insecurity), role demands (e.g., uncertainty about the requirements of their role), physical demands (e.g., inadequate lighting or heating at the workplace), and interpersonal demands (e.g., relationships with colleagues) (Quick et al., 1997). These demands may lead to the regular and prolonged experiences of stress and an increased allostatic load (Mücke, 2018) and are potential risk factors for mental health markers such as increased psychological distress (Dekker and Schaufeli, 1995). For example, individuals who are concerned about their job security will likely experience maladaptive effects on their wellbeing (Kler et al., 2015). Given that it is not possible to avoid stress as part of one’s goal-related pursuits, it is important to understand how individuals’ beliefs and responses to such demands may contribute to their mental health.

The ongoing demands associated with various occupations alone may not be a sufficient explanation as to why symptoms of psychological distress may manifest in employees. That is, whilst of course high demands are a risk factor for mental and physical health episodes (Suh and Punnett, 2022), research indicates that there are many psychosocial mediators of this relationship. For example, Gonzalez-Mulé and Cockburn (2021) used a 20-year time-lagged design with 3,148 individuals and found that perceived job control and cognitive ability buffered the positive relationship between job demands and poor mental health. They also found that physical and mental health mediated the moderated (by perceived job control and cognitive ability) job demands-mortality relationship. As such, whilst job demands were related to death via mental health, these relationships were bounded by perceived job control and cognitive ability. The indirect relationships between stress-related factors in the workplace was also highlighted by Abdullah and Al-Abrrow (2025) who highlighted that self-efficacy could attenuate job stress and burnout.

One potential psychological factor that might help explain psychological distress in working populations is irrational beliefs (e.g., Jones et al., 2021; Turner and Barker, 2015; Wood et al., 2021). Indeed, irrational beliefs have been identified as a trigger for symptoms of anxiety and depression, and in turn, this can also increase the likelihood of poor physical health (Vassou et al., 2021). In addition, a meta-analysis by Vîslă et al. (2016) revealed that irrational beliefs are consistently and positively associated with various types of psychological distress, such as general distress, anxiety, depression, anger, and guilt. In support, Turner M. et al. (2022) and Turner M. J. et al. (2022) demonstrated that employees holding high irrational beliefs reported poorer life satisfaction, lower work persistence, higher presenteeism and absenteeism, and higher intentions to quit, as well as greater symptoms of anger, anxiety, depression and stress compared to employees with low irrational beliefs. Irrational beliefs in the aforementioned extant research are codified specifically with the framework of Rational Emotive Behaviour Therapy (REBT; Ellis, 1957). In REBT, people’s experience of psychological distress is in part determined by their proclivity to hold and endorse extreme, illogical, and rigid beliefs or tacit schema concerning the self, the world, and others (Turner, 2022). That is, whilst the demands employees face are of course an important antecedent to psychological distress, the holding and application of irrational beliefs to those demands is likely to exacerbate this risk. As such, beliefs play a central role in psychological wellbeing, such that rational beliefs (i.e., flexible, logical, and non-extreme) underpin psychological wellbeing, whilst irrational beliefs (i.e., inflexible, illogical, and extreme) underpin psychological distress (Turner et al., 2019). Irrational beliefs are divided into four subcategories: demandingness (e.g., “I want, therefore I must…”), awfulizing (e.g., “It is awful if others do not approve of me), frustration intolerance (e.g., “I cannot stand not reaching my goals”), and self/other/life depreciation (e.g., “If I am not given opportunities, then it shows that I am not a worthwhile person”). An employee who believes that “I must perform well in my job and if I do not, it shows that I am a failure” holds demandingness and self-deprecation beliefs, and such cognitions will likely result in unhealthy negative emotions in employees (e.g., Turner and Barker, 2015).

Evidence continues to grow which associates irrational beliefs with a range of maladaptive outcomes for employees. For example, Popov and Popov (2013) found that irrational beliefs were positively related to psychological distress. In contrast, applying the core tenets of REBT may allow employees to interpret their thoughts and emotions in more adaptive ways, which may benefit their responses to adverse events in the workplace and beyond (Turner et al., 2024a; Turner et al., 2024b; Wood et al., 2021). Irrational beliefs are evidently problematic for work engagement and wellbeing, and thus, research that seeks to understand potential concomitents of irrational beliefs in employees is worthwhile. Overall, the REBT framework proposes that irrational beliefs are deleterious for a range of stress-related outcomes such as mental health (e.g., Vîslă et al., 2016), and this is supported by a growing weight of literature in adults across a range of occupational settings (e.g., Harris et al., 2006; Turner and Allen, 2018). However, the relationships between irrational beliefs and mental health is nuanced and there is a need to further explore how irrational beliefs may combine with other constructs in relating to mental health.

The ways in which irrational beliefs are associated with psychological distress is subject to ongoing research. Recent work has demonstrated that the relationship between irrational beliefs and poorer mental health may be mediated by a variety of factors. For instance, Mansell and Turner (2022) reported that irrational beliefs are related to psychological distress through self-confidence, whilst alternative mechanisms have been proposed including self-determined motivation (Turner M. et al., 2022; Turner M. J. et al., 2022), maladaptive schema (Turner et al., 2019) and negative automatic thoughts (Buschmann et al., 2018). Another proposed mechanism for the relationship between irrational beliefs and psychological distress are challenge and threat evaluations (Chadha et al., 2019; Meijen et al., 2020). Individuals may be predisposed to evaluate situations of personal significance as a challenge or a threat. When an individual evaluates a situation as having potential to harm their self-esteem or wellbeing, a threat evaluation may be evinced. In contrast, when individuals evaluate a meaningful situation to be an opportunity for success and they are confident their efforts will yield positive outcomes, the individual will likely evaluate the situation as a challenge (Lazarus and Folkman, 1984). Importantly, challenge and threat evaluations are associated with differing cognitions, emotions, and behaviours (Lazarus and Folkman, 1984). In the workplace, challenge evaluations are positively associated with job satisfaction (Podsakoff et al., 2007) and positive emotions (Searle and Auton, 2015). Contrastingly, threat evaluations are associated with higher psychological distress, exhaustion and reduced job dedication (Tuckey et al., 2015). Although some researchers have suggested that individuals could be challenged, threatened, challenged and threatened, or neither challenged or threatened by a particular stimulus (Uphill et al., 2019), other theoretical approaches have put forward the suggestion that such evaluations are dynamic (Meijen et al., 2020). This means that not only may individuals evaluate an important situation differently as either a challenge or as a threat over time, but their evaluation may alter *during* the situation. As such, opinion is divided as to whether challenge and threat evaluations are dichotomous or not. Overall, the approach-focus associated with challenge evaluations rather than avoidance-focus is said to result in more beneficial outcomes, such as enhanced work productivity (Casper et al., 2017) and performance (LePine et al., 2005).

How employees usually appraise stressors may be predicated on their dispositions (Troup and Dewe, 2002). Meijen et al. (2020) suggested that high irrational beliefs might predict greater threat evaluation, drawing upon correlational (Dixon et al., 2017), and path analytic findings (Chadha et al., 2019). To expand, Dixon et al. (2017) found that football coaches with greater irrational beliefs were more likely to appraise a stressor as a threat, rather than a challenge. Chadha et al. (2019) used path analysis and concluded that it is the combined effects of high irrational beliefs, low challenge evaluations and high threat evaluations that predicted negative emotion in the lead up to an important event. Much of the research concerning how irrational beliefs relate to challenge and threat emanates from sport literature. However, there may be some differences between athletic and work settings that could mean that this sport research cannot be translated into the work context. For example, in sports such as soccer, each week there is a competitive fixture usually in front of a large number of spectators. Most workplaces do not feature such an event, but competition is engaged in less directly and overtly on a daily basis internally (e.g., for promotion) and externally (i.e., in the market place). Also, athletes must contend with the physical danger of the training and competitive environment in a way that would be unusual for most workplaces. The workplace is more likely to host a number of diverse complex political, market-driven, and structural dynamics which will depend upon the sector and industry that need to be assimilated or contended with. However, in the workplace, employees can experience similar stressors to athletes and students, such as a lack of autonomy, unclear roles, and the need to achieve targets (Leka et al., 2003). Resultingly, challenge and threat evaluations are evinced, which can influence how employees experience stress, with challenge evaluations associated with less absenteeism (Gillman et al., 2023). With irrational beliefs also associated with worse mental health among workers (Santarpia et al., 2023), it seems that an individual who holds irrational beliefs is more likely to make threat evaluations and subsequently, they will likely experience negative emotions and anxiety. This may mean that the effects of irrational beliefs on mental health are magnified by threat evaluations, and therefore, it is important to investigate the roles of irrational beliefs in challenge and threat in working samples.

Another important determinant of employee performance and mental health at work is self-control (Pilcher and Bryant, 2016; Troup and Dewe, 2002), a factor that is also proposed as an outcome of challenge and threat evaluations (Jones et al., 2019). Self-control is used synonymously with other terms such as willpower and self-discipline, however, it is widely agreed that effortful self-regulation is a central tenet of self-control (Duckworth, 2011). Baumeister et al. (2007) describe self-control as an individual’s ability to alter their responses to an event, and in turn, such responses support the pursuit of relevant goals. These responses are deliberate, effortful, and often require an individual to make different responses or to refrain from taking a particular action (Baumeister et al., 2007) reflecting self-discipline and impulse control (Maloney et al., 2012). Those who possess high self-control are said to benefit from a range of adaptive outcomes such as work performance (De Ridder et al., 2012), greater challenge appraisals (Galla and Wood, 2015), and adaptive psychological states (Tangney et al., 2004). Low self-control may have a detrimental impact on cognitions, emotions and behaviour, such as anger (Kashif et al., 2020), and is reported to relate to negative physical and psychological health (De Ridder et al., 2012), such as depression (Özdemir et al., 2014), anxiety (Diestel and Schmidt, 2010), and worse performance (Schmeichel et al., 2003). Given the often-uncertain environment that employees operate in, adopting self-control strategies may be particularly useful because although it may not be possible to change the external event, individuals can instead focus on their own internal responses to the event (Troup and Dewe, 2002). This adaptive action aligns with the principles of REBT (Ellis, 1957) and may explain why possessing self-control is an important contributor to positive mental health.

**Figure 1:**
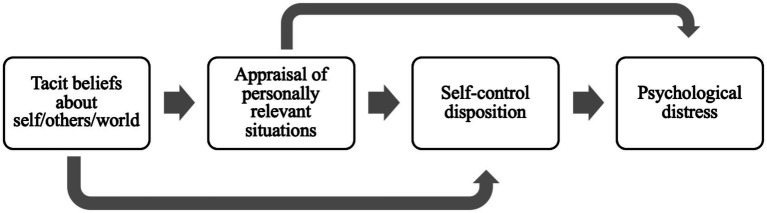
Theoretical model portraying the effects of beliefs, appraisal, and self-control on psychological distress.

In addition to the proposed relationship between irrational beliefs, self-control and mental health, it is possible that challenge and threat evaluations alongside irrational beliefs may influence self-control. In Figure 1 we model the proposed theoretical relationships between beliefs, appraisals, self-control, and psycholgical distress. This is in line with prominent contemporary theory, such as the revised theory of challenge and threat states in athletes (TCTSA-R; Meijen et al., 2020), and echoes recent research in which irrational beliefs and challenge and threat act together in their relationship to psychological distress (e.g., Chadha et al., 2024; Turner et al., 2024a; Turner et al., 2024b). To illustrate, an employee who believes that they are a worthless loser if they do not perform well (irrational belief) will likely evaluate their work-related events as more of a threat (e.g., Chadha et al., 2019). Subsequently, they are less likely to be able to sufficiently resource self-control efforts, since threat evaluation is indicative of insufficient resources to meet environmental demands (Jones et al., 2009). In other words, the lack of agency that one feels about being able to influence an upcoming task reflects low perceived control. Consequently, this may mean that an individual makes fewer efforts to regulate their responses to the situation.

Bakker and de Vries’ (2021) integrated job self-regulation demands-resources (JD-R) model indicates that increased job strain may bring about maladaptive self-regulation strategies. High job strain may lead to deficits in work performance, and when high job demands are persistent, employees may apply maladaptive self-regulation strategies like self-undermining (Bakker and Costa, 2014). This speaks to irrational beliefs, where self-undermining (or self-depreciation) is a common and pernicious belief made relevant by the experience of adversity (Turner, 2022). When high job demands are met with low job resources, combined with failed self-regulation, this may result in impaired performance (e.g., Roczniewska and Bakker, 2021) and poorer mental health and burnout (Bakker et al., 2023). Furthermore, the internalisation of self-statements is proposed to influence an individual’s self-control (Silverman and DiGiuseppe, 2001), and if these self-statements are undermining and maladaptive (e.g., irrational beliefs), then self-control is likely to be impaired. Thus, building on the work of Turner et al. (2019)and Chadha et al. (2024) in athletes, and Turner et al. (2024a) and Turner et al. (2024b) in university students, this confluence of irrational beliefs, challenge and threat evaluations, and self-control might provide a potent mixture of psychological factors that could be related to employee mental health.

**Figure 2:**
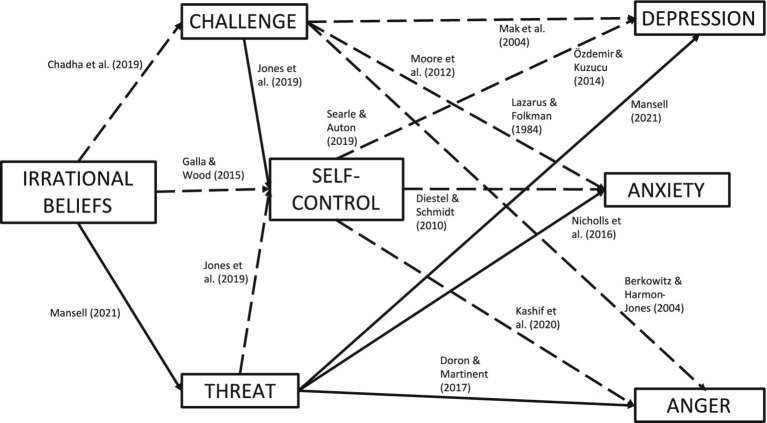
Hypothesized model. Dashed lines represent negative associations and unbroken lines represent positive associations. For visual simplicity controlling for age and lines of covariance between anger, anxiety and depressive symptoms are not shown.

Despite a range of evidence that demonstrates the relationships between irrational beliefs, challenge and threat evaluations, and self-control with mental health, there is little research that has explicitly investigated the confluence of these constructs. It seems that irrational beliefs relate to mental health through other constructs (e.g., Mansell, 2021), and previous research has highlighted the relationships between low self-control and poor mental health (De Ridder et al., 2012). Therefore, investigating the direct and indirect relationships between irrational beliefs, challenge and threat evaluations, and self-control in an integrative model may offer fresh insight to the factors that can influence the mental health of employees, and may inform methods to enhance employee mental health. The aims of the present study were to examine whether and to what extent irrational beliefs, challenge and threat evaluations, and self-control interact to influence markers of employee mental health (e.g., anger, anxiety and depression). In Figure 2 we offer an explication of Figure 1 which captures the hypothesised effects in the current study. Specifically, we hypothesised that higher irrational beliefs would be associated with greater threat evaluations (H1; Mansell, 2021), and lower challenge evaluations (H2; Chadha et al., 2019) and self-control (H3; Galla and Wood, 2015). Higher challenge and lower threat evaluations were hypothesised to be related with lower anger, anxiety and depression (H4; Moore et al., 2012), and higher self-control (H5; Mak et al., 2004; Searle and Auton, 2015). Finally, greater self-control was hypothesised to be related to lower anger, anxiety and depression (H6; Diestel and Schmidt, 2010). A secondary aim of the study was to assess whether there are any gender and age differences in any of the constructs. It was hypothesised that females would report greater irrational beliefs, threat evaluations, anxiety and depressive symptoms compared with males (H7), and that males would report greater challenge evaluations, self-control and anger than females (H8; e.g., Mansell, 2021). It was also hypothesised that age would be negatively associated with irrational beliefs, threat evaluations, anger, anxiety and depressive symptoms (H9), and positively associated with challenge evaluations and self-control (H10; e.g., Turner and Moore, 2016).

## Method

### Participants

Three hundred and fifty-one adults (*n* = 148 females, *n* = 164 males, *n* = 39 gender not provided, *M*age = 39.25 years, *SD* = 11.85) participated in the study. Although debate continues about optimal sample sizes for path analysis (Wolf et al., 2013), a rule of thumb of requiring a minimum of 10 participants per parameter is suggested (Jackson, 2003), which the present sample comfortably exceeds. Following ethical approval from the first author’s university ethics committee, participants were recruited through an online recruitment platform (Survio). Recruited participants represented ninety-six different occupations including managers (*n* = 61), teachers (*n* = 22) and lawyers (*n* = 19). The employees had taken part in their profession for an average of 11.68 years (*SD* = 9.74). Inclusion criteria were that employees were at least 18 years of age, proficient in English and were currently employed. Potential participants were provided with an information sheet about the study, then after providing informed consent, they completed an online questionnaire pack (via Qualtrics) containing demographic questions and a battery of psychometrics (c.15-min to complete) detailed below.

### Measures

*Irrational beliefs*. The irrational performance beliefs inventory (iPBI; Turner and Allen, 2018) was used to assess irrational beliefs (e.g., “It’s awful if others do not approve of me”). Across 28-items, participants rate the extent to which they agree/disagree with the statements on a five-point Likert-scale ranging from 1 (*strongly disagree*) to 5 (*strongly agree*). A mean score was calculated, with higher scores indicating a greater endorsement of irrational beliefs. In the present study, the Cronbach *α* coefficient indicated high levels of reliability (*α* = 0.94).

*Challenge and threat evaluations*. The Cognitive Appraisal Scale (CAS; Skinner and Brewer, 2002) was used to assess the extent to which individuals tend to appraise meaningful situations as a challenge (e.g., “Overall I expect that I will achieve success rather than experience failure”) or as a threat (e.g., “I feel that difficulties are piling up so that I cannot overcome them”). The CAS consists of 18-items and includes 8 items that assess challenge appraisal tendency and 10 items that assess threat appraisal tendency. Participants indicate the extent to which they agree or disagree with each statement by responding on a 6-point Likert scale ranging from 1 (*strongly disagree*) to 6 (*strongly agree*). For each subscale, the items were summed to produce separate challenge and threat appraisal scores. The Cronbach *α* coefficients in the present study were *α* = 0.83 (challenge) and *α* = 0.91 (threat), indicating high levels of internal reliability. The CAS is reported to provide valid and reliable challenge appraisal tendencies scores (e.g., Williams and Cumming, 2012).

*Self-control*. The Brief Self-Control Scale (BSCS; Tangney et al., 2004) was employed to assess individuals’ dispositional self-control. Responding to thirteen items (e.g., “I am good at resisting temptation”), participants are asked to consider the extent to which the statements reflect how they usually feel. Responses are made on a five-point Likert scale ranging from 1 (*not at all*) to 5 (*very much*). Nine of the items are reverse-scored and all items are then summed with higher scores indicating higher levels of self-control. The Cronbach’s *α* coefficient in the present study was *α* = 0.80, indicating good levels of internal reliability. Lindner et al. (2015) report that the BSCS is a valid and reliable measure of dispositional self-control.

*Anger, anxiety and depressive symptoms*. The State–Trait Personality Inventory (STPI; Spielberger et al., 1979) was used to measure dispositional anger, anxiety and depressive symptoms (e.g., “I feel nervous and restless”). The 40-item version of questionnaire was employed, with ten items each assessing trait anger, anxiety, depressive symptoms, and curiosity (not reported in this study). Participants were asked to indicate the extent to which they agree with each statement in relation to how they usually feel on a four-point Likert-scale ranging from 1 (*not at all*) to 4 (*very much so*). Scores were calculated by summing the ten items on each subscale to produce total scores for anger, anxiety and depressive symptoms. The Cronbach’s *α* coefficients in the present study were *α* = 0.89 (anger), *α* = 0.76 (anxiety) and *α* = 0.82 (depressive symptoms), indicating internal reliability. Previous studies have concluded that the STPI provides valid and reliable dispositional scores (e.g., Jacobs et al., 1988).

### Analytic strategy

#### Planned model

**Figure 3:**
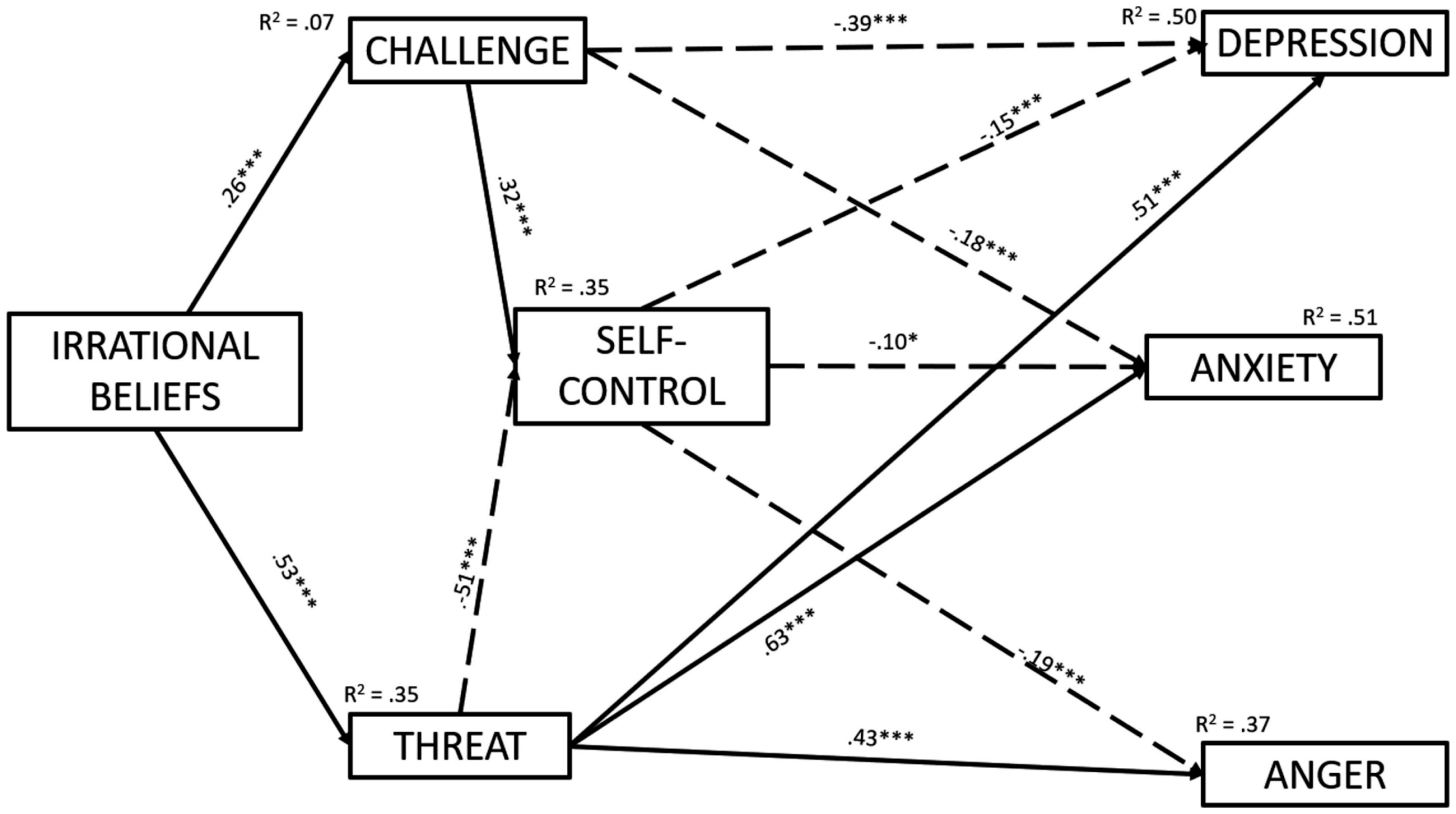
Path analysis testing the revised model for the effect of irrational beliefs, challenge and threat evaluations and self-control on anger, anxiety and depression. Note: Full lines denote positive relationships and dashed lines denote negative relationships. Numbers refer to standardized beta values. * = *p* < 0.05, ** = *p* < 0.01, *** = *p* < 0.001.

**Figure 4:**
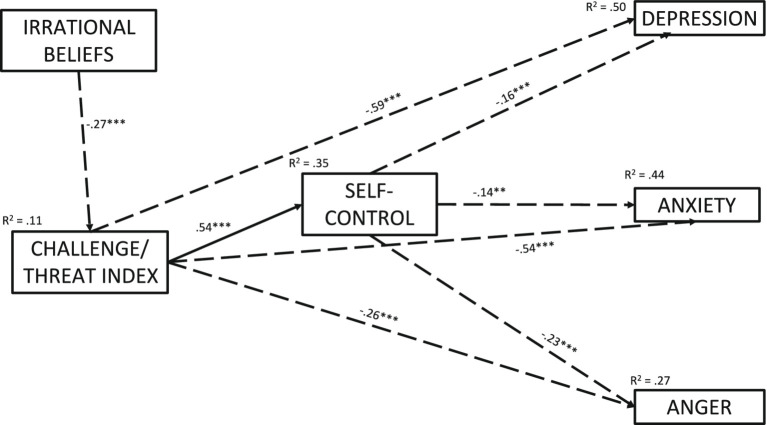
Path analysis testing the revised model for the effect of irrational beliefs, Challenge and Threat Evaluation Index (CTI), and self-control on anger, anxiety and depression.

**Figure 5:**
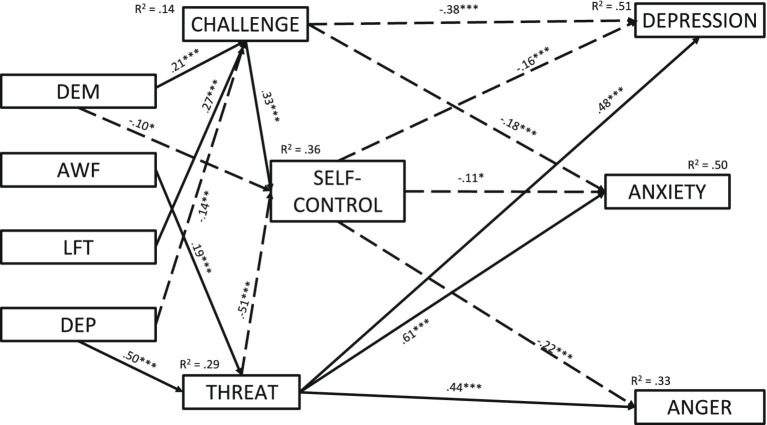
Path analysis testing the revised model for the effect of separate irrational beliefs, challenge and threat evaluations, and self-control on anger, anxiety and depression.

Data were screened for missing values and outliers in SPSS (IBM, version 28). No missing data were found. No univariate or multivariate outliers when using Mahalanobis distance at *p* < 0.001 (Tabachnick et al., 2013). The hypothesised model was tested using path analysis via SPSS AMOS (version 28) to determine direct and indirect effects between irrational beliefs, challenge and threat evaluations and self-control, and anger, anxiety and depression. This method of data analysis was chosen to simultaneously assess the relationships between the variables, whilst also examining the indirect effects in one integrated model (Byrne, 2010). Goodness of fit was examined using the chi square likelihood statistic ratio (χ2; Jöreskog and Sörbom, 1993), whilst the comparative fit index (CFI) and the Tucker Lewis fit index (TLI) were used as measures of incremental fit, with values of ≥ 0.95 and ≥ 0.90 exhibiting an excellent model fit (Hu and Bentler, 1999). To assess indices of absolute model fit, root mean square error of approximation (RMSEA) and standardized root mean square (SRMR) were employed, where criteria of ≤ 0.05 and ≤ 0.08 reflected excellent and adequate model fit, respectively, (Byrne, 2010). Similar measures of model fit were also used in other comparable studies (e.g., Chadha et al., 2019). In cases of poor model fit, regression weights and modification indices were examined to allow subsequent inclusion or exclusion of specific pathways in revised editions of the model (Byrne, 2010). Standardized regressions were reported for direct and indirect effects. Indirect effects were examined using 95% bias-corrected confidence intervals generated from bootstrapping of 1,000 samples. Given the range of ages within the sample, age was controlled for in the model, although for visual simplicity, it has been omitted from the Figures 2–5.

#### Exploratory model

The same approach was taken to assess the fit of an additional exploratory model to the data. Instead of assessing challenge and threat evaluations as two separate constructs, threat evaluations were subtracted from challenge evaluations (challeng score – threat score) to produce a challenge and threat evaluation index (CTI) based on the discrepancy between challenge and threat. Higher positive scores indicate greater challenge relative to threat, and higher negative scores indicate greater threat relative to challenge. Accordingly, this meant that the direct and indirect effects between irrational beliefs, CTI, and self-control, and anger, anxiety and depression were assessed.

## Results

### Differences in gender and relationships with age

A MANOVA indicated no significant gender differences in any of the variables. The relationship between age and the variables are displayed in Table 1. Age was shown to have a significant negative relationship with irrational beliefs, threat evaluations, anger, anxiety and depression, and a significant positive relationship with self-control. There was no significant correlation between age and challenge evaluations.

**Table 1:** Descriptive statistics and age correlations with variables.

Variable	Mean	SD	Pearson correlation	R^2^
Irrational beliefs	3.35	1.07	−0.208***	−0.043
Challenge evaluation	4.10	0.86	−0.048	−0.002
Threat evaluation	3.47	1.07	−0.283***	−0.080
CTI	−2.71	1.26	0.203***	0.041
Self-control	39.74	4.29	0.237***	0.056
Depression	20.84	5.67	−0.236***	−0.056
Anxiety	22.05	5.55	−0.202***	−0.041
Anger	22.27	6.84	−0.261***	−0.068

* = *p* < 0.05, ** = *p* < 0.01, *** = *p* < 0.001. CTI, challenge and threat evaluation index.

### Planned analyses

Path analysis revealed that the hypothesised model demonstrated an acceptable fit to the data, with the CFI and SRMR values suggesting an excellent fit *χ*^2^(3) = 41.95, *p* = 0.000, CFI = 0.97, TLI = 0.71, RMSEA = 0.19 (CI = 0.14, 0.25), SRMR = 0.03. Although the RMSEA value appears to suggest a poor model fit, this may be attributed to the simplicity of the model and the low degrees of freedom (*df* = 3). As such, the data can still be a good fit to the model (Kenny et al., 2015), even when one or more indicators are may suggest otherwise (Schermelleh-Engel et al., 2003). The TLI is also below the cut-off point for an excellent fit (≥ 0.90; Hu and Bentler, 1999), although it is recommended to report a suitable range of incremental and absolute model fit rather than reporting the ‘best’ numbers (Hooper et al., 2008). The standardized path coefficients for each individual path are displayed in Figure 3 and are accompanied by squared multiple correlation (R^2^) scores. Both offer support for the strength of fit of the data to the proposed model and demonstrate patterns largely consistent with study hypotheses.

Results of the indirect effects demonstrated that irrational beliefs was found to have a significant indirect effect through challenge and threat evaluations on self-control (*β* = −0.35, *p* = 0.002, 95% CI = −0.52, −0.19), anger (*β* = 0.44, *p* = 0.002, 95% CI = 0.32, 0.56), anxiety (*β* = 0.40, *p* = 0.002, 95% CI = 0.30, 0.50), and depression (*β* = 0.27, *p* = 0.002, 95% CI = 0.16, 0.37). Challenge evaluations had a significant indirect effect through self-control on anger (*β* = −0.49, *p* = 0.001, 95% CI = −0.84, −0.23), anxiety (*β* = −0.21, *p* = 0.016, 95% CI = −0.46, −0.04), and depression (*β* = −0.32, *p* = 0.001, 95% CI = −0.64, −0.13). Similarly, threat evaluations had a significant direct effect through self-control on anger (*β* = 0.63, *p* = 0.001, 95% CI = 0.33, 1.02), anxiety (*β* = 0.27, *p* = 0.020, 95% CI = 0.04, 0.53), and depression (*β* = 0.42, *p* = 0.001, 95% CI = 0.17, 0.73).

### Exploratory analyses

To explore whether challenge and threat evaluations as one construct rather than dichotomous variables, a similar model was tested (see Figure 4) using the CTI instead of separate challenge and threat evaluation scores. Path analysis revealed that this model demonstrated a mixed fit to the data *χ*^2^(3) = 89.91, *p* = 0.000, CFI = 0.92, TLI = 0.47, RMSEA = 0.29 (CI = 0.24, 0.34), SRMR = 0.09, with direct effect patterns commensurate with the model that included sepetate challenge and threat evaluation scores. Indirect effects demonstrated a significant indirect effect for irrational beliefs through the CTI on self-control (*β* = −0.27, *p* = 0.002, 95% CI = −0.39, −0.17), anger (*β* = 0.19, *p* = 0.002, 95% CI = 0.10, 0.28), anxiety (*β* = 0.22, *p* = 0.002, 95% CI = 0.14, 0.31), and depression (*β* = 0.25, *p* = 0.002, 95% CI = 0.16, 0.35). The CTI had a significant indirect effect through self-control on anger (*β* = −0.67, *p* = 0.001, 95% CI = −1.03, −0.35), anxiety (*β* = −0.32, *p* = 0.005, 95% CI = −0.56, −0.10), and depression (*β* = −0.38, *p* = 0.002, 95% CI = −0.64, −0.15).

We also tested a similar model to the planned model (Figure 3) that included each separate irrational beliefs type rather than a composite irrational beliefs scrore. Noting that irrational beliefs may relate to outcome variables in different ways (Mansell and Turner, 2022), the composite irrational beliefs measure was substituted for the four separate irrational beliefs (see Figure 5), and a model was tested to ascertain the fit of the data. Path analysis revealed that this model demonstrated a poor fit to the data *χ*^2^(20) = 784.04, *p* = 0.000, CFI = 0.62, TLI = 0.14, RMSEA = 0.33 (CI = 0.31, 0.35), SRMR = 0.26.

### Summary of results

In summary, Figure 3 was the model that best fitted the data. In this model, irrational beliefs were significantly and positively related to threat evaluation, but in contrast to the hypotheses, irrational beliefs were *positively* associated with challenge evaluation rather than *negatively*. In turn, challenge evaluation was found to be positively associated with self-control, and negatively associated with anxiety and depression. As hypothesised, threat evaluation was found to be positively associated with anger, anxiety and depression, and negatively associated with self-control. Also supporting the hypotheses, self-control was negatively associated with anger, anxiety and depression. Results of the indirect effects demonstrated that irrational beliefs were associated with self-control, anger, anxiety and depression through challenge and threat evaluations, and that challenge and threat evaluations were associated with anger, anxiety and depressive symptoms through self-control.

In the exploratory model, in which we used a challenge and threat evaluation index (CTI) instead of challenge and threat evaluations as separate constructs, although the model fit was not as good as the planned model. However, it is noteworthy that the model still contained several significant direct and indirect relationships between the variables. To illustrate, greater irrational beliefs are signifantly related to the lower CTI (less challenge, more threat). In turn, the CTI was significantly and negatively related to anger, anxiety, and depression, and significantly and positively related to self-control. Self-control was also significantly and negatively related to anger, anxiety, and depression. The direction of these relationships are in-line with H1-H6, albeit the relationship between irrational beliefs and self-control was non-significant.

## Discussion

The aims of the present study were to examine whether and to what extent irrational beliefs, challenge and threat evaluations, and self-control interact to influence employee mental health (e.g., anger, anxiety and depression). We aimed to propose and test a model in which employee mental health may be influenced by pathways incorporating irrational beliefs, challenge and threat evaluations, and self-control. Path analysis indicated that it is through threat evaluation and self-control that irrational beliefs may be related to employee mental health. That is, employees reporting higher irrational beliefs, alongside greater threat evaluations and poorer self-control, indicated worse mental health. In lay terms, if my engagement with work is such that I adopt rigid and extreme beliefs about work (e.g., “I must be treated with respect”), view stressors as threatening (e.g., “I cannot overcome my work demands”), and I am low in self-control (e.g., poor self-discipline), then I may be more at risk of presenting with poorer mental health symptoms, such as greater anger, anxiety, and depression. Due to the prevalence of poor mental health among adults (Baker, 2020) this work is important, and builds upon previous studies by indicating potential factors through which irrational beliefs might influence employees’ mental health. The present study extends the literature by offering challenge and threat evaluations and self-control as associates of employee mental health, but also as mechanisms through which irrational beliefs relate to employee mental health.

In support of H5, challenge (positively) and threat evaluations (negatively) were found to be directly associated with self-control. Although Jones et al.’s (2009) theory predicts that challenge and threat evaluations would influence self-control, research testing this hypothesis is sparse. Jones et al. (2009) predict that challenge would require less self-regulation (self-control) resources, whilst threat would require more self-regulation, making the argument that challenge is more efficient for the self-regulation of psychological states. In the current study, we found that challenge was related to greater self-control, whilst threat was related to poorer self-control. It could be that challenge evaluation allows for greater self-control because sufficient resources are in place for successful self-control, whereas for threat evaluation, sufficient resources are not in place. This link between challenge and threat, and self-control, might offer additional explanations as to why more challenge and less threat is consistently associated with superior performance in demanding cognitive and physical tests (e.g., Turner et al., 2012).

The present study demonstrates that challenge and threat evaluations were associated indirectly with mental health (i.e., anger, anxiety and depressive symptoms) through self-control. Whilst challenge and threat were also directly related to mental health, which supported H4, it is particularly through self-control that challenge and threat may exert this influence. In other words, an individual may tend to view stressors as a threat and this is likely to be associated with poorer mental health, but when combined with poor self-control, the deleterious effects on mental health are more pronounced. Poor self-control has been found to be related to worse mental health (e.g., Tangney et al., 2004), and threat evaluations underpin greater negative emotion and depressive symptoms (e.g., Mak et al., 2004), whilst challenge evaluations underpin greater positive emotions (Skinner and Brewer, 2002), lower anxiety (Moore et al., 2012), and reduced depressive symptoms (Mak et al., 2004). So, it is perhaps unsurprising that in combination, threat evaluation and poor self-control are related to worse mental health, and more correctly, that it is perhaps through self-control that threat evaluation influences mental health. Taken together, this emphasises the importance of promoting challenge evaluations as a route to enhancing workers’ mental health.

Whilst most study hypotheses were supported, there are some findings that do not conform to our predictions. Based on prior research (e.g., Chadha et al., 2019; Turner et al., 2024a; Turner et al., 2024b), irrational beliefs were proposed to relate negatively with challenge evaluations and positively with threat evaluations. Findings of the present study offer partial support for the hypothesis in that irrational beliefs were found to be positively associated with threat evaluations (H1), but a *positive* association between irrational beliefs and challenge evaluations was also revealed (H2). The positive relationship between irrational beliefs and challenge evaluations was unexpected but is plausible. Indeed, challenge and threat evaluations are not necessarily dichotomous in their nature and may relate to constructs in nuanced ways (Meijen et al., 2020). Having tested challenge and threat evaluations as a combined challenge and threat evaluations index (CTI) (see Figure 4), the data demonstrated a better fit to the model when assessing challenge and threat evaluations as separate constructs. However, similar significant relationships were uncovered in this model between challenge and threat evaluations as a combined construct (CTI) with self-control, anger, anxiety, and depression. Indeed, this means that either exploring challenge and threat evaluations as dichotomous constructs *or* using the CTI may hold value as researchers continue to understand the interplay between irrational beliefs and challenge and threat. In this case, our exploratory analysis suggests that assessing challenge and threat as separate variables supports a better model fit, which supports suggestions put forward by Uphill et al. (2019) in that individuals can experience challenge and threat at the same time. Indeed, Mansell (2021) also reported that challenge and threat appraisal tendencies related differently to irrational beliefs and mental health. To elaborate, Mansell (2021) reported that demandingness was positively related to challenge appraisal tendencies but not to threat appraisal tendencies, while challenge was positively related to vitality, but threat was not. Future researchers may wish to explore further the idea of the CTI to capture self-reported challenge and threat evaluations to capture challenge relative to threat. Indexing challenge and threat in this way is in line with how physiologic challenge and threat have been indexed using cardiac output and total peripheral resistance (e.g., Miller et al., 2021; Moore et al., 2012; Turner et al., 2012) but does perhaps remove some of the nuance of marking challenge and threat evaluations separately. Our expectation was that using a CTI would be more parsimonious for path analysis, but the less than acceptable model fit prohibits us from recommending the use of CTI in the way we have captured it in this paper.

As a new line of enquiry with a workplace sample, it might be possible that work pressure drives irrational beliefs, but that workers are able to hold challenge evaluations due to culturally learned responses to pressure or acclimatised patterns of appraisal. Despite the dysfunctional properties of irrational beliefs, the positive relationship between irrational beliefs and challenge evaluations may be explained by considering that irrational beliefs can offer a motivational function towards goal attainment (Turner, 2022). For example, the ‘musts’ associated with irrational beliefs (e.g., “I must be approved of”) may act as a call to action for employees, such as spending more time preparing their pitch for a contract or completing assigned work on time. To illustrate, those who possess irrational beliefs may endorse challenge evaluations captured by the measure we used to assess challenge, such as “*A challenging situation motivates me to increase my efforts*.” In other words, my demand to succeed, or be approved of, could furnish me with acute motivational gains that serve to boost my effort (Turner and Moore, 2016), such as spending more time on working towards a target. For some occupational workers, adopting such beliefs based on their perceived function may be, to them, a hallmark of a diligent and hard-working employee. But, despite the findings that demonstrate a positive relationship between irrational beliefs and an adaptive construct (i.e., challenge evaluations), caution should be urged regarding the positioning of irrational beliefs as useful. Given the raft of evidence that highlights the role of irrational beliefs in determining psychological distress across numerous population samples (Vîslă et al., 2016), it would be wise to promote alternative methods of increasing challenge evaluations, such as leader instructions (Turner et al., 2014), and reappraisal (Jamieson et al., 2018). The development of rational preferences may also be an effective route to promote challenge evaluations as they can still include a motivational focus (e.g., *“I really want to succeed”*), but without the risks to mental health posed by demandingness. Overall, although those holding strong irrational beliefs may benefit in the short term, they are less likely to harness motivational qualities that sustain long term success (e.g., Turner M. et al., 2022; Turner M. J. et al., 2022) and are likely to erode mental health (Turner M. et al., 2022; Turner M. J. et al., 2022).

In contrast to H3, irrational beliefs did not relate directly to self-control. As such, it seems that in the current sample, greater irrational beliefs are not associated with worse self-control. Irrational beliefs did relate indirectly to self-control through challenge or threat evaluations, adding weight to the idea that irrational beliefs may exert their influence on mental health risks factors through variables that are more proximal to mental health (Turner et al., 2019). This means that employees who possess high irrational beliefs and high threat evaluations are most at risk for experiencing low self-control. Our findings suggest that irrational beliefs and challenge and threat evaluations may combine to influence employees’ self-control, but that irrational beliefs are not the main player. It may be that irrational beliefs are most problematic to employee mental health when they are aligned with threat evaluation and poor self-control.

The present study also aimed to assess whether there were any relationships or differences between the variables based on employees’ demographic data. Contrary to H7 and H8, there were no observed gender differences in any of the variables. Although previous studies have noted gender differences (e.g., Mansell, 2021), the absence of any gender differences in this study reflects inconsistencies in gender differences in stress-related constructs in the workplace (Fida et al., 2023). In support of H9 and H10, age was shown to have a significant negative relationship with irrational beliefs, threat evaluations, anger, anxiety and depression, and a significant positive relationship with self-control. There was no significant correlation between age and challenge evaluations. In general, it appears that individuals develop more helpful thinking strategies as they grow older, and this may facilitate better mental health (Tomaka and Magoc, 2021). Although we collected data on occupation, we aimed to assess the variables across a general working population rather than examine in differences between occupations. Future research may wish to investigate whether targeting specific occupations would replicate our findings from a general sample.

### Strengths, limitations, and future research

Turning to the strengths and limitations of the present study, the deployment of path analysis to account for multiple relationships simultaneously allowed us to assess direct and indirect relationships on multiple outcomes, and provides a clear visual representation of such relationships (Byrne, 2010). However, it should also be noted that the path analysis consists of cross-sectional data, and thus the pathways are atemporal. This means that we do not propose that these cognitions necessarily occur in the order that appear in the path model, or indeed that cause-effect is occurring. However, we do include an appropriate sample size to undertake our analyses, and future studies may wish to use the path model presented here as a basis for experimental and longitudinal work, such as assessing whether targeting reductions in irrational beliefs and threat evaluations can lead to increased self-control, and subsequent mental health gains in occupational employees. A strength of the present study is the gender balance of the participants, although future research may also wish to collect data regarding ethnicity and socio-economic status. A limitation of the study is that the wide range of occupations does not allow us to examine whether there are differences between occupations in the constructs, or to properly account for occupation type in our model testing. Our findings may also be subject to common method bias given that the same measure is used to collect data from multiple sources (Kock et al., 2021). Finally, a limitation is the mixed consistency of the model fit indices. Despite excellent model fit indices (CFI, SRMR), RMSEA and TLI were poor and caution should be urged with our findings. Accordingly, future research could use our proposed model as a template for further exploration in occupational settings.

In the current study, we limit our assessment of mental health markers to self-reported symptoms of anger, anxiety, and depression, but it may also be of value to assess work-based outcomes such as absenteeism (Diestel and Schmidt, 2010) and turnover intention (Podsakoff et al., 2007). Previous research has indicated that, for example, greater irrational beliefs are related to greater absenteeism, productivity, and turnover intention (Turner M. et al., 2022; Turner M. J. et al., 2022), but has not utilised challenge and threat and self-control in these endeavours. As such, although there is some indication that irrational beliefs, challenge and threat, and self-control are separately important for markers of work engagement, their combined and interactive influence is currently unknown.

The results of the present study offer some potential ways forward for promoting individual mental health in the workplace. For example, given the apparent importance of irrational beliefs, REBT-informed programs can be developed and delivered in workplaces to help employees weaken their irrational beliefs, such as those exemplified in previous research (e.g., Barker and Turner, 2015). Indeed, the beliefs captured in the iPBI are relevant in all occupational settings because regardless of their profession or seniority, workers will experience the adversities captured by the iPBI frequently, such as rejection, failure, and disapproval. When workers endorse irrational beliefs about such adversities, their mental health is at risk. Accordingly, due to the ubiquity of workplace adversity, our findings could be generalised across various occupational settings. Also, promoting challenge and discouraging threat can be achieved using programs that apply cognitive behavioural techniques such as reappraisal (Jamieson et al., 2018). Noting the findings from the present study, this would likely enhance self-control. As an antecedent of challenge evaluations, developing self-efficacy in employees could be one route to mitigating the effects of stress in the workplace (Abdullah and Al-Abrrow, 2025). Challenge-informed language applied through leadership (Turner et al., 2024a; Turner et al., 2024b; Slater et al., 2018) and the creation of a socially supportive environment (Meijen et al., 2020) could also enhance workers’ mental health. To expand, given the relationships between challenge and threat evaluations and mental health proposed in the present study, leaders in occupational settings may wish to adopt the 3Rs approach (Reflecting, Representing and Realising; Haslam et al., 2020) to enhance relational identity between the leader and team. Leaders may also wish to cultivate circumstances for social support among employees to enhance challenge evaluations (Ben-Zur and Michael, 2007) and offset low self-control (Pilcher and Bryant, 2016). Leadership development programs could include education on the importance of adopting rational language in the workplace, while it is recommended that at an organizational level, wellbeing initiatives are proactively employed to support workers’ mental health (Phythian et al., 2023). Adopting such initiatives may not only benefit individual workers but may have economic and societal benefits by increasing productivity and reducing factors such as absenteeism – whether workers are experiencing clinical or subclinical symptoms of poor mental health (LaMontagne et al., 2014).

## Conclusion

In this study we used path analysis to assess the relationships between irrational beliefs, challenge and threat evaluations, and self-control, and key mental health indicators in employees. Largely supporting the hypothesis, results suggest that irrational beliefs may operate through challenge and threat evaluation and self-control in influencing employee mental health, and that threat evaluation appears to be a particularly powerful risk factor towards poorer mental health. Results from the indirect effects also suggest that relationships between challenge and threat evaluations and psychological distress may occur through self-control. This model adds to the literature by helping us to understand the relative predictive roles of irrational beliefs, challenge and threat evaluations, and self-control on employee mental health, and by elucidating the link between irrational beliefs and self-control. Speaking to the latter, irrational beliefs were only indirectly, and not directly, associated with self-control via challenge and threat evaluation. Whilst the importance of challenge and threat evaluation for employee mental health needs to be more fully studied, there are indications that work-based programs that can lower irrational beliefs and threat evaluation may have a positive impact upon self-control and subsequent mental health.

## Data Availability

The raw data supporting the conclusions of this article will be made available by the authors, without undue reservation.
